# Have Regulatory Efforts to Reduce Organophosphorus Insecticide Exposures Been Effective?

**DOI:** 10.1289/ehp.1104323

**Published:** 2012-01-17

**Authors:** Alison L. Clune, P. Barry Ryan, Dana Boyd Barr

**Affiliations:** Department of Environmental Health, Rollins School of Public Health, Emory University, Atlanta, Georgia, USA

**Keywords:** biomonitoring, dialkylphosphate metabolite, FQPA, NHANES, organophosphorus insecticide

## Abstract

Background: The Food Quality Protection Act (FQPA) was signed into law in 1996 to strengthen the regulation of pesticide tolerances in food. Organophosphorus (OP) insecticides were the first group of pesticides reviewed by the U.S. Environmental Protection Agency (EPA) under the new law.

Objective: Our goal was to determine whether urinary concentrations of dialkylphosphate (DAP) metabolites of OP pesticides declined between the National Health and Nutrition Examination Survey (NHANES) III and NHANES 1999–2004.

Methods: Using mass spectrometry–based methods, we analyzed urine samples from a nationally representative sample of 2,874 adults 20–59 years of age in NHANES 1999–2004 and samples from a non-nationally representative sample of 197 adult participants for NHANES III (1988–1994) for six common DAP metabolites of OP pesticides.

Results: Median urinary DAP concentrations decreased by more than half between NHANES III and NHANES 2003–2004. Reductions of about 50%–90% were also observed for 95th percentile concentrations of five of the six metabolites. Frequencies of detection (FODs) decreased in all six metabolites (< 50% reduction). On average, median and 95th percentile concentrations and FODs showed a larger decrease in diethylphosphate metabolites than dimethylphosphate metabolites.

Conclusions: Human exposure to OP insecticides as assessed by urinary DAP concentrations has decreased since the implementation of the FQPA, although we cannot be certain that U.S. EPA actions in response to the FQPA directly caused the decrease in DAP concentrations.

Until the mid-1990s, pesticide regulation relied on the Federal Insecticide, Fungicide, and Rodenticide Act (FIFRA 1947) and the Federal Food, Drug, and Cosmetic Act (FFDCA 1938), which were not totally congruent in their language. To make the regulatory efforts stronger and more consistent, the Food Quality Protection Act (FQPA) of 1996 (FQPA 1996) was signed into law on 3 August 1996. The FQPA amended FFDCA and FIFRA to include cumulative and aggregate exposure risk assessments in derivative food tolerance levels. In addition, special consideration was to be given to exposures among children (FQPA 1996). Because of their common mode of toxicity as potent acetylcholinesterase inhibitors, the U.S. Environmental Protection Agency (EPA) selected organophosphorus (OP) insecticides as the first class of pesticides for reassessing food tolerances. The chemical-specific assessments of nearly all of the OP pesticides were completed by August 2006, resulting in the cancellation of the residential uses of all but a few OP pesticides (U.S. EPA 2006a).

Since passing the FQPA, total use of OP pesticides in the United States increased between 1996 and 1999 from 75 to 91 million pounds per year, mainly because of the U.S. Department of Agriculture cotton boll weevil eradication program, but decreased to 46 million pounds by 2004 (Grube et al. 2011). Residential use of OP pesticides may have declined more quickly, largely because of the voluntary cancellation of residential uses of chlorpyrifos and diazinon in 2000 (U.S. EPA 2006b, 2006c). Although the phaseout of most residential uses of chlorpyrifos and diazinon was not completed until 2005 (U.S. EPA 2006b) and 2004 (U.S. EPA 2006c), respectively, some reports suggest that use of these OP insecticides declined shortly after the announcement of the cancellations (Johnson et al. 2011; Whyatt et al. 2003, 2004).

To assess the effectiveness of the U.S. EPA’s enforcement of the FQPA, however, human exposure to OP pesticides must also be assessed. Because exposure typically occurs through multiple routes and because dominant routes of exposure vary (Egeghy et al. 2005; Pang et al. 2002), assessing exposure to OP pesticides is not a trivial process. Measurement of exposure markers has been used to estimate the absorbed dose of OP pesticides in several matrices (Berman et al. 2011; Whyatt et al. 2003, 2004), but particularly in urine. Although some studies use specific urinary OP metabolites to characterize exposure to several OP pesticides (Arcury et al. 2007, 2010; Barr et al. 2005; Fenske et al. 2002; Lu et al. 2006), specific metabolites have not been identified for many OP pesticides (Wessels et al. 2003) and therefore cannot represent cumulative OP pesticide exposure. Alternatively, many studies of both children and adults have quantified six urinary dialkylphosphate (DAP) metabolites common to several OP pesticides in occupational (Azaroff and Neas 1999; Mills and Zahm 2001; Yucra et al. 2006), paraoccupational (Koch et al. 2002; Mills and Zahm 2001), and background exposure settings (Aprea et al. 1996; Barr et al. 2004; Bouchard et al. 2011; Bradman et al. 2005; Castorina et al. 2003; Engel et al. 2007; Eskenazi et al. 2004; Heudorf and Angerer 2001; Lu et al. 2001; Sexton et al. 2011; Ye et al. 2008, 2009; Young et al. 2005). These measurements provide no specific information about the pesticide(s) to which a person was exposed and may represent exposure to both the pesticide itself and its environmental degradate(s). However, urinary DAP metabolite measurements may provide useful information about cumulative exposure to OP pesticides as a class, as approximately 75% of the U.S. EPA–registered OP pesticides break down to form one to three of these six DAP metabolites (Wessels et al. 2003).

Barr et al. (2011) recently published urinary DAP metabolite concentrations, specifically those of dimethylphosphate (DMP), dimethylthiophosphate (DMTP), dimethyldithiophosphate (DMDTP), diethylphosphate (DEP), diethylthiophosphate (DETP), and diethyldithiophosphate (DEDTP), among 2,874 adults 20–59 years of age from the general U.S. population for 1999 to 2004. These data were collected from the National Health and Nutrition Examination Survey (NHANES) 1999–2004 [Centers for Disease Control and Prevention (CDC) 2009a] during three 2-year collection cycles and are representative of the civilian, noninstitutionalized U.S. population, stratified by age, sex, and race/ethnicity. Here, we report data from a random subset (*n* = 197) of a convenience sample of adults (≥ 18 years of age) from NHANES III (1988–1994), also called the “call-back” cohort, samples that were collected before implementation of the FQPA. Participants ≥ 18 years of age were asked if they would like to participate in the environmental sampling component (sometimes referred to as the Priority Toxicant Reference Range Study) of NHANES. If they agreed, they were called back for an appointment the following day, when a single blood draw and a single urine sample were collected for analysis of volatile organic compounds (VOCs) and pesticide metabolites, respectively. Since then, these samples have been used to measure phthalates, mercapturic acid metabolites of VOCs, and other environmentally related chemicals/metabolites. These data are not representative of the U.S. population, but they provide a useful picture of potential OP pesticide exposure among adults before the FQPA was implemented. By comparing these data sets that were generated in the same laboratory, we can evaluate the effectiveness of the FQPA in reducing OP pesticide exposure as measured by the reduction of urinary concentrations of DAPs between the NHANES III and NHANES 1999–2004.

## Methods

The National Center for Health Statistics of the CDC (NCHS/CDC) conducted the NHANES studies. NHANES is designed to measure the health and nutrition status of the civilian, noninstitutionalized U.S. population (CDC 2009a). NHANES participants were selected based on their age, sex, and racial/ethnic background through a complex statistical process using the most current census information. For this study, we analyzed data from urine samples from an approximate one-third random subset of participants in NHANES 1999–2004 (Barr et al. 2011). Urine specimens were collected from participants 20–59 years of age (*n* ≈ 2,900 depending on the analyte) during one of three daily examination periods. Sociodemographic information and medical histories of survey participants and their families were collected during the household interview.

To evaluate pre-FQPA concentrations of DAP metabolites among adults, we analyzed 197 urine samples collected as part of NHANES III, which was conducted from 1988 to 1994. These were randomly selected from a convenience sample of survey participants ≥ 18 years of age who agreed to provide urine samples for environmental chemical analysis. Although this sampling was not representative of the U.S. population, it provided a reasonable reference range for DAP concentrations resulting from background exposures during the time period.

The NCHS/CDC Institutional Review Board reviewed and approved the study protocols from NHANES III and NHANES 1999–2004. Informed written consent was obtained from all participants at the time of sample collection.

*Laboratory methods.* During the physical examinations, urine specimens were collected from participants, aliquoted, and stored cold (2–4°C) or frozen until shipment. To determine urinary creatinine concentrations, we used an automated colorimetric method based on a modified Jaffe reaction (Jaffe 1886) on a Beckman Synchron AS/ASTRA clinical analyzer (Beckman Instruments, Inc., Brea, CA) at the University of Minnesota Medical Center. Samples collected for OP pesticide measurements were shipped on dry ice to the CDC National Center for Environmental Health. Urine samples were analyzed for DAP metabolites of OP pesticides using the methods of Bravo et al. (2002, 2004). The 2002 method was used to analyze samples collected in NHANES III and NHANES 1999–2000. The 2004 method was used for subsequent analyses. The two methods were shown to agree (Barr et al. 2011). Briefly, 4 mL urine was spiked with an isotopically labeled internal standard mixture, then concentrated to dryness using an azeotropic codistillation with acetonitrile or lyophilization. The dried residue was dissolved in acetonitrile, and the DAPs were derivatized to their respective chloropropyl esters using 1-chloro-3-iodopropane and potassium carbonate. The solution containing the chloropropyl esters was concentrated and then analyzed using gas chromatography–positive chemical ionization–tandem mass spectrometry. The DAP metabolites were quantified using isotope-dilution calibration. The analytic limits of detection (LODs) were 0.5–0.58 μg/L for DMP, 0.18–0.5 μg/L for DMTP, 0.08–0.2 μg/L for DMDTP, 0.2 μg/L for DEP, 0.09–0.1 μg/L for DETP, and 0.05–0.1 μg/L for DEDTP, with the ranges indicating differences among NHANES cycles. When analyzed, LOD achievable samples for NHANES III were slightly higher than LOD achievable samples for NHANES 1999–2004. Consequently, we did not perform formal statistical testing of differences in frequencies of detection (FODs). The LOD differences were considered when making general observations regarding the FOD. Both laboratories and methods were certified according to the Clinical Laboratory Improvement Amendments (CLIA 1988) guidelines.

*Statistical analysis.* SAS software (version 9.1.3; SAS Institute Inc., Cary, NC) and SUDAAN software (version 9.0.1; Research Triangle Institute, Research Triangle Park, NC) produced median and 95th percentile estimates. SUDAAN uses sample weights to account for the unequal probability of selection. Geometric means were not calculated, as the FOD was < 60% for most analytes, making such estimates unreliable. For a concentration or estimate < LOD, we used a value equal to the LOD divided by the square root of two (Hornung and Reed 1990). Although techniques for imputing values < LOD have been debated in the literature (Caudill et al. 2007; Chen et al. 2011; Curwin et al. 2010; Gillespie et al. 2010; Jain and Wang 2008; Jain et al. 2008), we used this imputation technique consistent with the CDC National Report on Human Exposure to Environmental Chemicals (CDC 2009b). Other imputation techniques may produce slightly different estimates. Trends in urinary DAP concentrations were examined by calculating the relative change in median and 95th percentile concentrations, and the absolute change in the FOD between the NHANES III call-back cohort and the NHANES 2003–2004 cycle. Diethyl-substituted (DE) metabolites, including DEP, DETP, and DEDTP, originate from different OP pesticides than dimethyl-substituted (DM) metabolites, including DMP, DMTP, and DMDTP, so trends in urinary DE and DM concentrations were also examined separately.

To specifically examine the impact of the voluntary cancellation of chlorpyrifos and diazinon in residential settings, we examined trends in the summed median concentrations of DEP and DETP, metabolites of numerous OP pesticides including the commonly used chlorpyrifos and diazinon. Volume-based median concentrations of DEP and DETP were converted to molar concentrations and summed to examine trends in the cumulative concentration of these metabolites. We considered this a valid calculation because all of the U.S. EPA–registered pesticides that produce DEP also produce DETP, and vice versa, and because concentrations of DEP and DETP were found to be correlated in the NHANES 1999–2004 sample (Barr et al. 2005). Analysis of variance with an *F*-test for significance (*p* < 0.05) was used to assess the temporal trend between the two NHANES monitoring periods. Because inter- and intraindividual variation in both metabolism and intake may alter the proportion of each metabolite per individual, the summed measure may be a better overall indicator of diethyl-substituted OP insecticide exposure.

## Results

**Figure 1:**
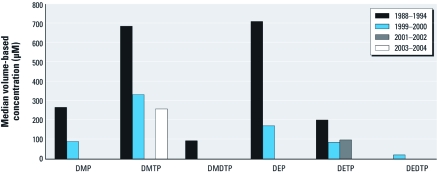
Median volume-based concentrations of the six DAP metabolites of OP insecticides in the NHANES III (1988–1994) and 1999–2004.

**Figure 2:**
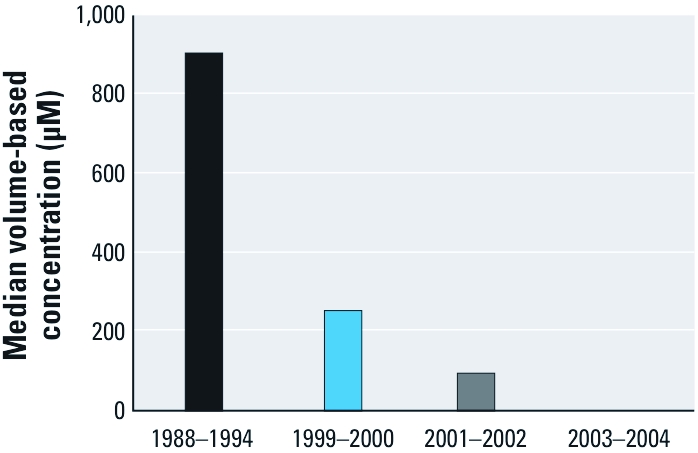
Summed median concentrations of DEP and DETP metabolites in NHANES III (1988–1994) and 1999–2004.

Table 1 shows the summary statistics derived from adult participants of the NHANES III call-back cohort and NHANES 1999–2004. All median DAP concentrations decreased significantly between NHANES III and NHANES 1999–2004 with the exception of DEDTP, which was the most infrequently detected DAP. On average, median concentrations of DAP metabolites decreased by 84.0% (range 63.1–98.5%) (Figure 1). Median DE concentrations demonstrated a greater average decrease than did median DM concentrations (92.1% and 73.9%, respectively). Although the median DETP concentration did not show a consistent downward trend across all NHANES cycles, when DEP and DETP concentrations were summed within each cycle, a consistent downward trend in the medians was observed (Figure 2). Five of six 95th percentile DAP metabolite concentrations decreased by an average of 70.1% (range 47.2–90.9%), with the exception of DMP, which increased by 5.2%. As with median metabolite concentrations, 95th percentile DE concentrations decreased more than those of DM metabolites, on average (74.4% and 63.6%, respectively). All DAP metabolites had a lower FOD in the NHANES 2003–2004 sample than in the NHANES III cohort despite improved LODs in NHANES 2003–2004. The FOD decreased by 34.4% (range 13.3–46.2%), on average. DE metabolites demonstrated a larger average decrease in the FOD than did DM metabolites (39.8% and 29.1%, respectively).

**Table 1:** Median and 95th percentile estimates of DAP metabolites of OP insecticides in the NHANES III (1988–1994) and 1999–2004.

Analyte/ survey years*a*		LOD (µg/L)	Volume based (µg/L)			Creatinine adjusted (µg/g creatinine)			FOD (%)
			*n*	Median	95th percentile	Median	95th percentile		
DMP															
	1988–1994		0.5		192		2.07*		13.4**		2.14*		25.2*		90.4
	1999–2000		0.58		814		0.68		9.7		0.76		14.6		50.9
	2001–2002		0.5		1,121		< LOD		11.5		< LOD		11.5		45.7
	2003–2004		0.5		938		< LOD		14.1		< LOD		13.5		46.1
DMTP															
	1988–1994		0.18		189		4.83*		65.4*		4.49*		42.6		86.8
	1999–2000		0.18		813		2.30		39.0		1.90		47.5		61.3
	2001–2002		0.4		1,121		< LOD		30.0		< LOD		25.2		46.9
	2003–2004		0.5		715		1.78		28.5		1.67		30.4		73.5
DMDTP															
	1988–1994		0.08		194		0.57*		17.4		0.47*		19.0		65.5
	1999–2000		0.08		814		< LOD		17.0		< LOD		20.5		47.3
	2001–2002		0.1		1,122		< LOD		4.90		< LOD		6.33		30.8
	2003–2004		0.1		925		< LOD		5.07		< LOD		5.71		35.8
DEP															
	1988–1994		0.2		184		4.6*		26.9*		5.00*		61.5*		92.4
	1999–2000		0.2		814		1.10		11.0		0.86		12.1		67.6
	2001–2002		0.2		1,122		< LOD		10.4		< LOD		7.34		48.1
	2003–2004		0.1		921		< LOD		14.2		< LOD		11.9		46.2
DETP															
	1988–1994		0.09		196		1.16*		17.9*		1.40*		13.8*		84.7
	1999–2000		0.09		814		0.49		2.0		0.25		2.75		52.3
	2001–2002		0.1		1,122		0.54		3.83		0.49		4.69		67.8
	2003–2004		0.2		918		< LOD		2.65		< LOD		2.82		47.2
DEDTP															
	1988–1994		0.05		197		< LOD		2.42*		0.12		2.15*		43.2
	1999–2000		0.05		814		0.09		0.90		0.08		0.86		54.4
	2001–2002		0.1		1,118		< LOD		0.83		< LOD		1.03		18.8
	2003–2004		0.1		938		< LOD		0.22		< LOD		0.4		7.51
**a**Survey years 1988–1994 were a part of the NHANES III survey call-back cohort, and the calculated percentile estimates are not representative of the U.S. population. *Represents a statistically significant (*p* < 0.05) decrease observed from NHANES III and all subsequent NHANES cycles. **Represents a significant (*p* < 0.05) decrease from NHANES III to NHANES 1999–2000 only.															

## Discussion

Urinary DAP metabolites of OP pesticides in adults in the U.S. population have decreased substantially since passing the FQPA. Although rather modest decreases in FOD for these metabolites can be attributed to improved quality assurance procedures with concomitant improvement in LOD stability, the decline in urinary DAP metabolites is likely related to the mitigation efforts of the U.S. EPA in phasing out most residential uses and limiting other uses of OP pesticides such as chlorpyrifos and diazinon. In addition, urinary concentrations of DEP and DETP, metabolites of the voluntarily withdrawn OP pesticides chlorpyrifos and diazinon, have consistently declined in the U.S. population since the removal of these pesticides from the residential market. Of the eight OP pesticides remaining in the residential market and considered in the 2006 cumulative risk assessment (i.e., acephate, bensulide, dichlorvos, disulfoton, malathion, naled, tetrachlorvinphos, trichlorfon), five form DM metabolites, one forms DE metabolites, and two do not form any DAP metabolites. Five of the six residential-use OP pesticides that form DM metabolites form only DMP, and a relative increase in the use of these OP pesticides could explain the small increase in 95th percentile urinary DMP concentrations. Median DM concentrations, however, decreased overall, despite the fact that the majority of OP pesticides currently registered for residential use form DM metabolites. This observation suggests that the use of OP pesticides as a class has declined and that the decrease is not solely attributable to the cancellation of registrations for DE-producing OP pesticides.

Although our data support an overall decline in OP pesticide exposures, it is important to note that these observations are subject to several key limitations.

First, the populations studied here were not equivalent before and after the passage of the FQPA. That is, although the adults in the NHANES 1999–2004 sample were nationally representative, the adults in the NHANES III sample were not. To our knowledge, however, no other nationally based sample of adult urinary DAP concentrations existed before implementation of the FQPA, and only a few, small studies measured DAP metabolites in adults during this period in the United States (McCurdy et al. 1994; Simcox et al. 1999). At minimum, the NHANES III call-back cohort was sampled at the national level from a representative population. Although it is not representative itself, the NHANES III call-back cohort provides the best available estimation of OP pesticide exposure in the general U.S. population before implementation of the FQPA. It is worthy of note that the still-registered chemicals in the original selection of chemical-specific assessments issued between 2000 and 2006 are evaluated. Further changes in registration are likely based on the as yet unfinished OP pesticide cumulative risk assessment. Age differences between participants in the two studies may also be of concern; the NHANES 1999–2004 sample consisted of adults in a more restricted age group (20–59 years) than the NHANES III sample (≥ 18 years of age). In the 1990 U.S. Census, adults 20–59 years of age made up 76.3% of all adults ≥ 20 years of age (Meyer 2001). We expect that this is a reasonable estimate for the makeup of the original NHANES III sample. We do not know the age distribution of participants in the NHANES III call-back cohort, but we do not anticipate that age would have influenced participation. Thus, it is reasonable to assume that the age distribution of the call-back cohort reflected that of the general population. Given the 1990 census data, we estimate that about one quarter of the participants in the NHANES III call-back cohort was not 20–59 years of age. Barr et al. (2011) demonstrated that adults ≥ 60 years of age tended to have higher urinary DAP concentrations than did younger adults in NHANES 2001–2004. Assuming that a similar age effect was observed in the NHANES III call-back cohort, the urinary DAP estimates presented here may overestimate those of the adult population 20–59 years of age during 1988–1994 because of the influence of samples from older adults.

Second, DAP metabolites in urine may arise from exposure to OP pesticides or exposure to preformed DAPs from environmental degradation of OP pesticides (Krieger et al. 2003; MacIntosh et al. 1999). In a California study, DAPs were present in larger quantities than OP pesticides on the surface of 60% of 153 fruits and vegetables sampled (Zhang et al. 2008). DAP degradates have also been found in fresh fruit juices (Lu et al. 2005) and household dust (Weerasekera et al. 2009). According to some estimates, 70% or more of urinary OP metabolite concentrations may be attributable to exposure to preformed DAPs in the environment (Morgan et al. 2005; Wilson et al. 2003). Animal evidence suggests that preformed DAPs are not further altered in the body (Timchalk et al. 2007). Urinary DAP metabolites have only a few minor sources aside from OP pesticides (Barr and Needham 2002), and we have no reason to expect that the proportion of urinary DAPs representing exposure to parent OP pesticides changed between NHANES III and NHANES 2003–2004. Therefore, the presence of DAPs in urine can be expected to track relative changes in exposure to OP pesticides, despite providing an overestimate of true exposure. Consequently, the observed decreases in urinary DAP concentrations suggest that human exposure to OP pesticides has declined in the United States over the past decade.

Third, we cannot be certain that U.S. EPA actions in response to the FQPA directly caused the observed decrease in DAP metabolite concentrations. We know that pesticide use decreased overall in the United States and that OP pesticides accounted for a diminishing share of total insecticide use (67% and 40% in 1994 and 2004, respectively) between NHANES III and NHANES 2003–2004 (Grube et al. 2011). These national-level changes could have resulted from the restricted availability of OP pesticides under the FQPA. Alternatively, a shift in consumer preference away from OP pesticides or an overall trend toward decreasing pesticide use that would have continued in the absence of the FQPA could explain our results. That DE metabolites were observed to decrease more than DM metabolites, however, suggests that the withdrawal of most residential uses of chlorpyrifos and all residential uses of diazinon, as well as the small number of DE-producing OP pesticides remaining for residential use as a result of the FQPA, directly affected the decline in exposure to OP pesticides. It should be noted that exposure to OP pesticides continues, as a substantial number of crop-specific agricultural uses remain. With the substantial reduction in residential use, attention is being directed toward ascertainment of the potential risks from the diet, particularly for children. We note further that there has been a substantial decrease in the urinary concentration of DAP metabolites associated with chlorpyrifos and diazinon, whose use in indoor environments has been substantially curtailed. This suggests that residential use was a primary pathway of exposure for these chemicals prior to their phaseout, as little change in agricultural use of these OP pesticides has occurred.

## Conclusions

Despite the limitations of our data, urinary DAP concentrations in NHANES III and NHANES 1999–2004 suggest that the FQPA has successfully contributed to a relative decline in exposure to OP pesticides. Specifically, urinary concentrations of DE metabolites, including those of the voluntarily withdrawn OP pesticides chlorpyrifos and diazinon, have consistently declined more than urinary concentrations of DM metabolites formed from other OP pesticides. Although the two studies are not directly comparable because of differences in methodology thereby limiting the quantification of the differences in national exposures, they offer the best data currently available to investigate trends in urinary DAP concentrations and the effectiveness of regulation of these products.
